# *α*-2-Macroglobulin in Saliva Is Associated with Glycemic Control in Patients with Type 2 Diabetes Mellitus

**DOI:** 10.1155/2015/128653

**Published:** 2015-03-03

**Authors:** Juan Pablo Aitken, Carolina Ortiz, Irene Morales-Bozo, Gonzalo Rojas-Alcayaga, Mauricio Baeza, Caroll Beltran, Alejandro Escobar

**Affiliations:** ^1^Departamento de Patología y Medicina oral, Facultad de Odontología, Universidad de Chile, 8380492 Santiago, Chile; ^2^Instituto de Investigación en Ciencias Odontológicas, Facultad de Odontología, Universidad de Chile, 8380492 Santiago, Chile; ^3^Departamento de Odontología Conservadora, Facultad de Odontología, Universidad de Chile, 8380492 Santiago, Chile; ^4^Hospital Clínico, Servicio de Gastroenterología, Facultad de Medicina, Universidad de Chile, 8300456 Santiago, Chile

## Abstract

*Background*. Subjects with type 2 diabetes mellitus (DM2) require an adequate glycemic control to avoid diabetic complications. Currently, saliva biomarkers are used as a diagnostic tool and can be indicative of the degree of progression and control of various diseases. Several studies indicate that *α*-2-macroglobulin levels are elevated in diabetic patients. *Methods*. 120 subjects with DM2 were enrolled and classified into two groups according to their glycemic control (percentage of glycated hemoglobin-A1c (HbA1c), <7% adequate glycemic control group; >7% inadequate glycemic control group). The relationship between *α*-2-macroglobulin levels from saliva samples and HbA1c was subsequently evaluated. *Results*. We found a positive correlation between *α*-2-macroglobulin and HbA1c (*r* = 0.778 and *P* < 0.0001). Area under the receivers operating characteristic (ROC) curve of *α*-2-macroglobulin indicated a positive discrimination threshold of *α*-2-macroglobulin (AUC = 0.903, CI 95%: 0.847–0.959, *P* < 0.0001) to diagnose glycemic control. *Conclusions*. Our data strongly suggest that the level of saliva *α*-2-macroglobulin is an indicator for the degree of glycemic control in diabetic patients and represents a promising alternative method to evaluate this parameter.

## 1. Introduction

Type 2 diabetes mellitus (DM2) is a global crisis that threatens the health and economy of all nations, particularly developing countries [1]. DM2 patients have at least a twofold risk of mortality compared to healthy people, and comorbidity with both macro- and microvascular disease is substantial [2]. Glycemic control in DM2 is important to manage the disease. Currently the best characterized parameter available is glycated hemoglobin-A1c (HbA1c), as it gives us an overview of an individuals' glycemic control from the previous 4 months [3]. However, this procedure can be invasive and associated with patient aversion of venous blood sample control. In this regard, there is a need to identify alternate screening places and other types of biological samples to evaluate glycemic control in DM2. Some attempts such as collecting gingival crevicular blood (GCB) to test HbA1c have been shown to be feasible and acceptable for diabetes screening in periodontal patients over finger stick blood [4, 5]. Until now testing HbA1c at saliva level is not possible, because saliva in normality does not contain blood. Nevertheless, there is sufficient evidence indicating that the majority of compounds found in blood are also present in saliva [6]. Given that obtaining samples is easier and noninvasive and has a lower economical cost, saliva represents a promising alternative auxiliary method of diagnosis that may be advantageous over current methods. At present, saliva biomarkers indicate the existence or the risk of developing a disease, as well as the response to a particular therapy [7].

Several studies have demonstrated that *α*-2-macroglobulin (A2MG) levels are increased in the blood of DM1 and DM2 patients with complications such as diabetic retinopathy [8–10]. Moreover, A2MG levels in plasma have been correlated with HbA1c percentages [11]. Rao et al. characterized the salivary proteome in DM2 and found that A2MG was differentially increased in the saliva of patients with disease progression and those with a prediabetic to diabetic state [12].

Thus far, there have been no studies examining saliva as a source of the A2MG biomarker and its association with glycemic control in DM2. Therefore, we investigated the relationship between saliva A2MG and HbA1c in patients with DM2.

## 2. Methods

### 2.1. Study Design and Population

The study was performed in agreement with the Helsinki Declaration [13] and approved by the Ethics Committee of the Faculty of Dentistry, University of Chile. All patients signed an informed consent. From July 2013 to December 2013, we prospectively enrolled 120 type 2 diabetes mellitus patients, women (67.5%) and men (32.5%), aged from 31 to 79 years (average age 61.6 years). Patients who are with rheumatic diseases, irradiated, pregnant, with terminal illnesses, with neurological damage, and with acute inflammatory processes in the mouth were excluded from the current study. All the subjects were recruited from the Diabetes Association of Chile (ADICH). In this study, the detailed demographic and clinical data were collected from all subjects. Patients with HbA1c levels <7% were classified as having adequate glycemic control and those with levels >7% were classified as having inadequate glycemic control [14].

### 2.2. Biomarker Measurements

Levels of HbA1c were measured using the Variant II brand team Bio-Rad (Bio-Rad, Inc., Hercules, CA), certified to the National Glycohemoglobin Standardization Program of the United States [15]. Human *α*-2-macroglobulin levels in saliva samples were determined using enzyme-linked immunosorbent assay (Human Alpha 2-Macroglobulin DuoSet, R&D Systems, Minneapolis, USA) according to the manufacturer's instructions. Briefly, samples and standard protein were added on plates previously coated with a capture antibody A2MG and serum blocked and incubated for 2 h. Plates were then washed 4 times and biotinylated anti-human A2MG detection antibody was added and incubated for 2 h. After 4 washes, plates were treated with streptavidin-HRP for 30 minutes and washed 5 times. Finally, following 10 minutes of incubation proteins were detected using TMB Solution, the reaction was stopped using Stop Solution, and plates were read at 450 nm. Concentrations shown are based on an internal standard curve generated for each experiment.

### 2.3. Statistical Analysis

Quantitative variables were expressed as mean ± standard error (SE). Nonparametric Spearman correlation test was used to assess the correlation between HbA1c and A2MG. Receivers operating characteristic (ROC) curves were constructed at the most discriminating cutoff values aimed at documenting the predictive power of A2MG in saliva to diagnose inadequate glycemic control. A *P* value of less than 0.05 was considered as statistically significant. Statistical studies were carried out with Stata 11.0 software and GraphPad Prism V5.0 software.

## 3. Results

### 3.1. Baseline Characteristics

The study population consisted of 120 type 2 diabetic patients (81 (67.5%) female and 39 (32.5%) male) with an average age of 61.6 ± 10.1 (ranging from 31 to 79 years). The average body mass index (BMI) was 28.9; 45 subjects (37.5%) displayed a HbA1 level lower than 7% and were thus considered as having adequate glycemic control, while 75 subjects (62.5%) exhibited a HbA1c level higher than 7% and hence were deemed as having inadequate glycemic control. Furthermore, in addition to having DM2, 74 patients were also found to be hypertensive (HT) (Table 1).

### 3.2. Correlation between Saliva Levels of A2MG and HbA1 Percentage

Using Spearman correlation analysis we found a correlation between saliva levels of A2MG and HbA1c percentage (*r* = 0.7748 and  *P* < 0.0001) in patients with DM2 (Figure 2).

### 3.3. Utility of Saliva Levels of A2MG for Predicting Severity of Inadequate Glycemic Control in Type 2 Diabetic Patients

**Figure 1:**
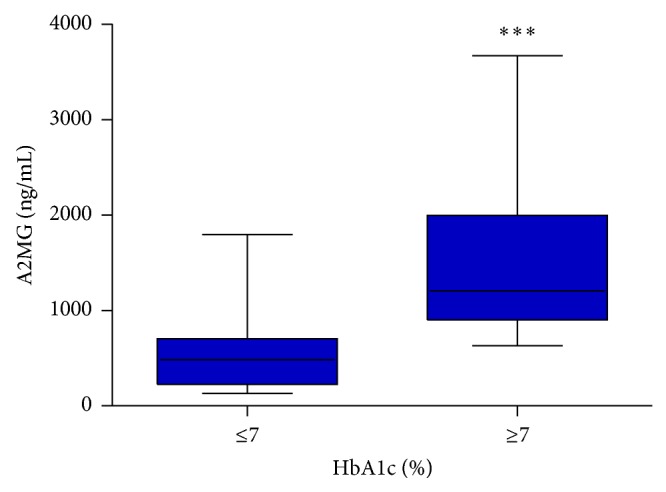
Box-plot showing the comparison of saliva levels of A2MG between patients with adequate and inadequate glycemic control (HbA1c percentages <7% and >7%).

**Figure 2:**
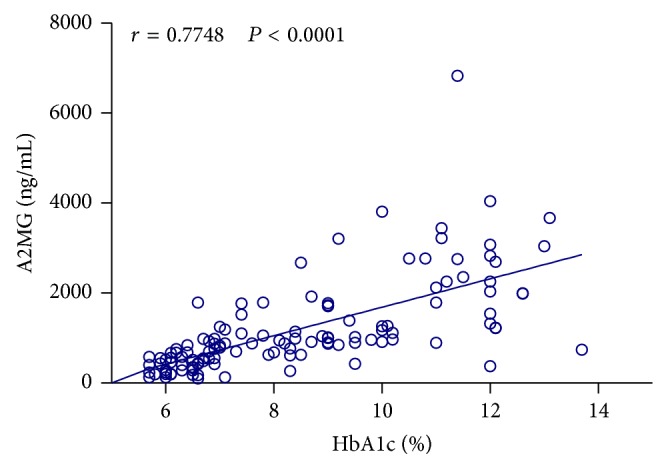
Scatter diagram showing the association between HbA1 percentage and saliva levels of A2MG based on Pearson's correlation analysis.

**Figure 3:**
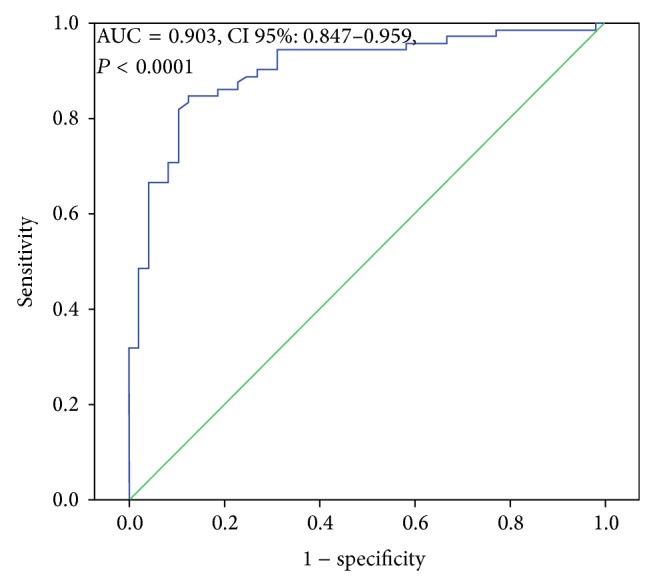
ROC curve of saliva levels of A2MG in DM2 patients with inadequate glycemic control. Receiver operating characteristic (ROC) curves displayed a positive discrimination threshold of A2MG in saliva to diagnose inadequate glycemic control in subjects with DM2.

Saliva levels of A2MG were found to be significantly higher in patients with inadequate glycemic control compared to those with adequate glycemic control (Figure 1) (*P* < 0.0001). Thereby, the area under the ROC curves indicated a positive discrimination threshold of A2MG (AUC = 0.903, CI 95%: 0.847–0.959, *P* < 0.0001) for patients diagnosed with inadequate glycemic control (Figure 3). The optimal cutoff value of saliva levels of A2MG to predict inadequate glycemic control was 840 ng/mL (sensitivity of 81.9% and 1 − specificity of 89.6%).

## 4. Discussion

In this study, we have shown a strong association between glycemic control and saliva levels of A2MG in subjects with DM2. The results obtained with the ROC curve indicate that A2MG could be used as a diagnostic method for distinguishing inadequate glycemic control subjects with high specificity. Our results are in line with those of Rao et al., who detected higher concentrations of A2MG in blood and saliva of prediabetic state subjects compared to healthy controls [12]. To date, no study had reported a correlation between saliva levels of A2MG and percentage of HbA1. Studies performed by Takada and Soman et al. [9, 16] have shown similar results; however, the levels of A2MG were detected only in blood. Presently, emerging biotechnologies are advancing our knowledge of biomarkers and there is sufficient evidence establishing that the majority of compounds found in blood may also be found in saliva [6]. Since saliva contains many possible serum-derived proteins in addition to secretions from major and minor salivary glands [17], it is conceivable that A2MG could be transferred by exocytosis from blood to saliva when this protein is present at high levels in plasma. Furthermore, studies indicate that A2MG is elevated in the blood of patients with DM1 and DM2, particularly in those with other complications due to diabetes, such as chronic renal failure or nephrotic syndrome [9].

We observed a positive association between total protein concentration in saliva and A2MG concentration in saliva (*r* = 0.24 and  *P* < 0.05) (data not shown), which is in line with Bencharit et al., who found that greater inadequacy in glycemic control is associated with an increase in total protein concentration detected in saliva [18]. However, we cannot disregard the possibility that the highest concentration of protein could be due to the disintegration of salivary mucin observed in diabetic patients.

Periodontal status is also related with saliva levels of A2MG; in fact one study has described that A2MG levels in crevicular fluid are significantly higher in patients with aggressive periodontitis compared to those with chronic periodontitis [19]. In our study, we only evaluated the periodontal status by an oral clinical examination; however this diagnosis requires complementary exams such as radiography [20]. In this regard, we may assume that patients with inadequate glycemic control included in our study may also present aggressive periodontitis [21] that may contribute to the high levels of A2MG detected in this group. However, Border et al. previously established that edentulous subjects with DM2 had high concentrations of salivary biomarkers associated with inflammatory processes, including A2MG [22]. To evaluate the main source of A2MG we performed a differential determination of A2MG levels in saliva from the parotid and sublingual glands of subjects with adequate and inadequate glycemic control (Figure S1 in Supplementary Material available online at http://dx.doi.org/10.1155/2015/128653). Our results demonstrate that at least 30% of A2MG levels detected in whole saliva originate from these major salivary glands. This result combined with those reported by Eliasson and Carlén supports the idea that A2MG levels measured in saliva are supplied primarily by the major and also minor salivary glands, as minor gland saliva is known to be involved in whole saliva composition [23].

Glycemic control in DM2 has been shown to reduce microvascular disease and improve quality of life [24]. Today, alternate screening places to evaluate glycemic control in DM2 using HbA1c from GCB have been evaluated in periodontal patient taking advantage of dental visit [25]. Our findings indicate a positive correlation between levels of A2MG in saliva and percentage of HbA1c showing a possible complementary strategy for diabetes screening using saliva. As showed by Strauss et al. [4], we also believe that the dental visit is an opportune site for diabetes screening by levels of A2MG in saliva.

Findings of our study are limited because it represents only a one-center study with a single measurement. To solve this issue, a future research requires using periodic measurements over a period of at least one year (every 3-4 months) and assessment in response to therapy.

In conclusion, our work indicates a positive correlation between levels of A2MG in saliva and percentage of HbA1c and suggests that measurement of A2MG in saliva represents a promising alternative method to evaluate glycemic control and consequently avoiding comorbid associated pathologies.

## Supplementary Material

Levels of A2MG in saliva from parotid and sublingual gland were found to be significantly lower than whole saliva (p< 0.01) in both type 2 diabetic patients groups with adequate and inadequate glycemic control, representing at least 30% of A2MG levels detected in whole saliva (Figure S1).

## Figures and Tables

**Table 1:** Baseline demographic and clinical characteristics based on the percentage of glycosylated hemoglobin.

	HbA1c <7% (*n* = 45/37.5%)	HbA1c >7% (*n* = 75/62.5%)	Total (*n* = 120/100%)
Male	12 (26.6%)	27 (36%)	39 (32.5%)
Age (years)	60.2 (10.2)	62.4 (10.1)	61.6 (10.1)
BMI	29.0 (4.2)	28.8 (4.5)	28.9 (4.4)
HT	29 (64)	45 (60)	74 (61.6)

BMI: body mass index; HT: hypertension; HbA1c: glycosylated hemoglobin. There were no statistically significant differences in any parameters.
