# Supplementary material for: α-2-Macroglobulin in Saliva Is Associated with Glycemic Control in Patients with Type 2 Diabetes Mellitus
Source: Dis Markers. 2015 Mar 3;2015:128653. doi: 10.1155/2015/128653 (PMC4363888; doi:10.1155/2015/128653)

Supporting Information

Figure S1 Scatter diagram showing the association between total protein concentration and A2MG concentration in saliva based on Pearson's correlation analysis.

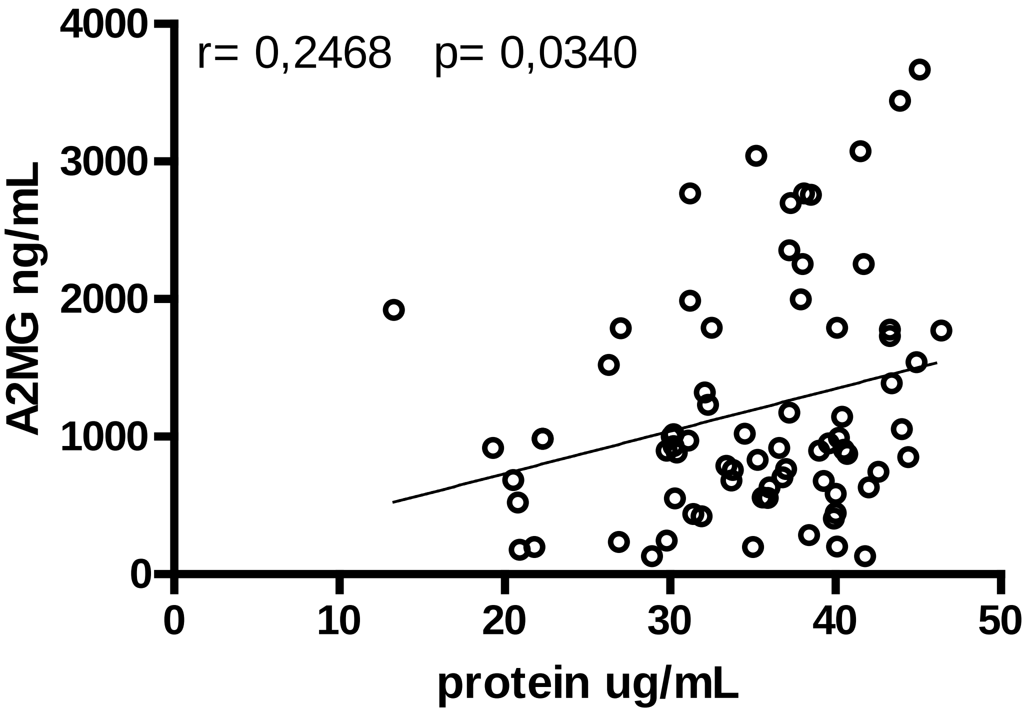

Figure S2 Bar graph showing the comparison of saliva levels of A2MG from whole saliva, parotid saliva and sublingual saliva between patients with adequate and inadequate glycemic control. Data represent a n=50; bars indicate SEM; ** $p < 0.01$. *** $p < 0.001$ indicates differences between patients with HbA1c percentage <7% and > 7%.

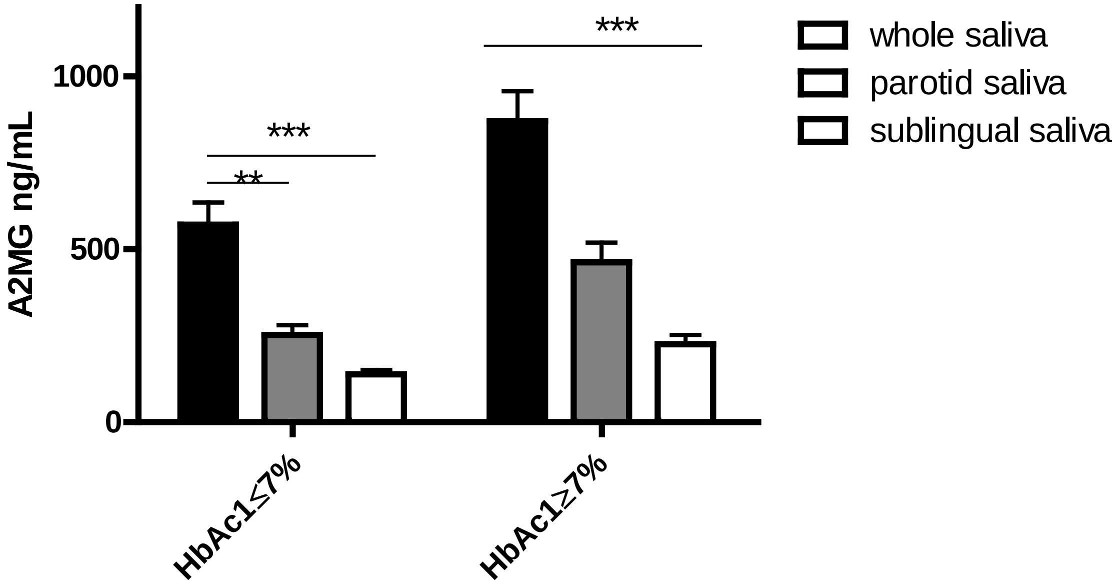

Supplement: Supplementary file 1 — Levels of A2MG in saliva from parotid and sublingual gland were found to be significantly lower than whole saliva (p< 0.01) in both type 2 diabetic patients groups with adequate and inadequate glycemic control, representing at least 30% of A2MG levels detected in whole saliva (Figure S1). [file 128653.f1.pdf]
